# Supplementary material for: Realist evaluation of the impact of the research translation process on health system sustainability: a study protocol
Source: BMJ Open. 2022 Jun 12;12(6):e045172. doi: 10.1136/bmjopen-2020-045172 (PMC9196166; doi:10.1136/bmjopen-2020-045172)
Supplement: Author's manuscript [file bmjopen-2020-045172.draft_revisions.pdf]

BMJ Open is committed to open peer review. As part of this commitment we make the peer review history of every article we publish publicly available.

When an article is published we post the peer reviewers' comments and the authors' responses online. We also post the versions of the paper that were used during peer review. These are the versions that the peer review comments apply to.

The versions of the paper that follow are the versions that were submitted during the peer review process. They are not the versions of record or the final published versions. They should not be cited or distributed as the published version of this manuscript.

BMJ Open is an open access journal and the full, final, typeset and author-corrected version of record of the manuscript is available on our site with no access controls, subscription charges or pay-per-view fees (http://bmjopen.bmj.com).

If you have any questions on BMJ Open's open peer review process please email info.bmjopen@bmj.com

BMJ Open

Realist evaluation of the impact of the research translation process on health system sustainability: a study protocol

Journal:	*BMJ Open*
Manuscript ID	bmjopen-2020-045172
Article Type:	Protocol
Date Submitted by the Author:	07-Oct-2020
Complete List of Authors:	Mosedale, Abby; Curtin University, School of Public Health<br>Hendrie, Delia; Curtin University, School of Public Health<br>Geelhoed, Elizabeth; Telethon Kids Institute<br>Zurynski, Yvonne; Macquarie University, Australian Institute of Health Innnovation<br>Robinson, Suzanne; Curtin University, Health Policy Management
Keywords:	Organisation of health services < HEALTH SERVICES ADMINISTRATION & MANAGEMENT, PUBLIC HEALTH, Quality in health care < HEALTH SERVICES ADMINISTRATION & MANAGEMENT

SCHOLARONE™
Manuscripts

I, the Submitting Author has the right to grant and does grant on behalf of all authors of the Work (as defined in the below author licence), an exclusive licence and/or a non-exclusive licence for contributions from authors who are: i) UK Crown employees; ii) where BMJ has agreed a CC-BY licence shall apply, and/or iii) in accordance with the terms applicable for US Federal Government officers or employees acting as part of their official duties; on a worldwide, perpetual, irrevocable, royalty-free basis to BMJ Publishing Group Ltd ("BMJ") its licensees and where the relevant Journal is co-owned by BMJ to the co-owners of the Journal, to publish the Work in this journal and any other BMJ products and to exploit all rights, as set out in our licence.

The Submitting Author accepts and understands that any supply made under these terms is made by BMJ to the Submitting Author unless you are acting as an employee on behalf of your employer or a postgraduate student of an affiliated institution which is paying any applicable article publishing charge ("APC") for Open Access articles. Where the Submitting Author wishes to make the Work available on an Open Access basis (and intends to pay the relevant APC), the terms of reuse of such Open Access shall be governed by a Creative Commons licence – details of these licences and which Creative Commons licence will apply to this Work are set out in our licence referred to above.

Other than as permitted in any relevant BMJ Author's Self Archiving Policies, I confirm this Work has not been accepted for publication elsewhere, is not being considered for publication elsewhere and does not duplicate material already published. I confirm all authors consent to publication of this Work and authorise the granting of this licence.

For peer review only

TITLE

Realist evaluation of the impact of the research translation process on health system sustainability: a study protocol

Corresponding author: *Ms Abby Mosedale*. School of Public Health, Curtin University, Perth, Australia.

Email: abby.mosedale@curtin.edu.au

Address: Kent Street, Bentley, Perth

Western Australia, 6102

Tel: +61 8 9266 9266 Fax: +61 8 9266 2255

Co-authors:

Dr Delia Hendrie.

School of Public Health, Curtin University, Perth, Australia.

Professor Elizabeth Geelhoed.

Telethon Kids Institute, Perth, Australia

Associate Professor Yvonne Zurynski

Australian Institute of Health and Innovation, Macquarie University, Sydney, Australia

Professor Suzanne Robinson.

School of Public Health, Curtin University, Perth, Australia.

Funding statement: This work is supported by the National Health and Medical Research Council Partnership Centre for Health System Sustainability (ID: 9100002). In addition the Western Australian Department of Health provides financial support for the Research Translation Projects program.

Word count: 3874 words

For peer review only - http://bmjopen.bmj.com/site/about/guidelines.xhtml

ABSTRACT

Introduction: Sustainability at a system level relates to the capacity of the system to be able to service the ongoing health needs of the population. It is a multifaceted concept encompassing both the affordability and efficiency of a system and the system's ability to adapt and change.

To address issues that currently threaten health system sustainability, health care leaders, policy makers, clinicians and researchers are searching for strategies that capitalise on new research and technologies while ensuring the delivery of safe, value-based care. Often these strategies are assessed in terms of the intervention being sustainable rather than contributions to the sustainability of the system as a whole. We present a realist evaluation of a research translation program to understand how the research translation process contributes to health system sustainability through the implementation of interventions funded to deliver service improvement and value-based health care.

Methods and analysis:

Underpinned by the realist evaluation framework, this research will be executed in three phases: (1) developing the Initial Program Theory (IPT) of the research translation process; (2) testing the program theory through case study analysis; and (3) theory refinement and consolidation. The evaluation uses a case example of a research translation program, chosen for its representation of a microcosm of the broader health system and the heterogeneity of service improvement activities taking place within it. Across the three phases, analysis of data from program documentation, interviews and focus groups will draw on the context (C), mechanism (M), outcome (O) formula that is core to realist evaluation. In addition, system dynamic methods will capture the feedback loops and complex relationships amongst IPT and CMO configurations.

Ethics and Dissemination: Curtin University Human Research Ethics Committee, Western Australia, approves this study. Results will be published in scientific journals, and communicated to respondents and relevant partners.

For peer review only - http://bmjopen.bmj.com/site/about/guidelines.xhtml

STRENGTHS AND LIMITATIONS OF THIS STUDY

- This is the first study to evaluate the impact of the research translation process on health system sustainability.
- Adopting a realist methodology will allow contextual factors and mechanisms to be identified that influence how the research translation process can contribute to health system sustainability.
- A novel feature of this study is the application of system dynamics modelling within the realist evaluation framework, capturing the complexity and feedback relationships between context, mechanism and outcome.
- Using a Delphi method to establish consensus on implementation and evaluation recommendations for a research translation program will facilitate perspectives to be gained from a broad group of stakeholders.
- Evaluation of one research translation program as a case study may affect generalisability of study findings to the research translation process more generally.

INTRODUCTION

Health systems around the world are facing challenges relating to rising healthcare expenditure and the effectiveness and efficiency of the system to deliver high value, safe healthcare. A number of complex interdependent factors are contributing to these challenges. An aging population, rising chronic disease, public expectations, and a lack of value-consciousness amongst healthcare consumers and providers are the major factors driving growing demand for healthcare [1,2]. These issues are occurring against a cultural backdrop that is resistant to change, often driven by behaviours associated with vested interests, and incentives that do not promote value or transparency [3]. These challenges, pressures and behaviours raise questions about capacity of the system to deliver affordable, cost-effective outcomes to the population over time, which is often referred to as the sustainability of the system [4]. Research translation is an essential process for ensuring health systems have ongoing capacity to service the health needs of a population and address challenges through the integration of cost effective interventions based on new research and technology.

Western Australia (WA) is typical of other jurisdictions in Australia facing challenges that threaten the long-term sustainability of its health system. The demand for health services has grown substantially in recent years, along with health expenditure, yet outcomes in population health and acute care in WA have not improved at the same rate [3]. With major health issues such as increasing obesity, an aging population, chronic disease, mental health and inequalities in health outcomes

For peer review only - http://bmjopen.bmj.com/site/about/guidelines.xhtml

For peer review only

across the WA population, the sustainability of the WA health system is a growing concern for healthcare leaders and policy makers. The WA Department of Health, along with healthcare leaders, policy makers, clinicians and researchers around the globe, acknowledge the potential of research evidence to improve health outcomes and optimise resource use [3,5,6]. In 2007 the former State Health Research Advisory Council within the WA Department of Health, introduced the Research Translation Projects (RTP) program [7]. The RTP program aims to cultivate and translate evidence for a sustainable health system through the support of industry driven, high-quality research projects that have the potential to deliver improved cost effectiveness and/or efficiencies to the health system while maintaining or improving patient outcomes. Internal evaluations of the RTP program have focused primarily on research outputs such as the number of publications, changes to practice guidelines and additional research funding obtained. Whilst assessment of research outputs is important, the RTP program's contribution to sustainability is not well understood. Therefore, the project presented here, aims to explain how the RTP program contributes to health system sustainability through the implementation of research translation interventions funded to deliver service improvement and value-based health care. By adopting a realist evaluation framework, this project will in addition to the evaluation of the RTP program, aim to contribute to a more generalizable understanding of the relationships between the process of research translation, intervention sustainability and sustainability from a whole of system perspective.

BACKGROUND

Sustainability at a system level relates to the capacity of the system to be able to service the health needs of the population into the future[4]. It is a multifaceted concept that encompasses both the affordability and efficiency of a system, and the system's ability to adapt and change [2].

To address issues that currently threaten health system sustainability, healthcare leaders, policy makers, clinicians and researchers are searching for strategies that capitalise on new research and technologies while ensuring the delivery of safe, value-based care. Many of these strategies involve implementing new interventions and programs as research translation interventions [8].

Evaluating the impact of these interventions has become increasingly important as governments, research institutions and research funders are held to account for the funds they spend [5]. One aspect of evaluation is the sustainability and spread of interventions, that is their sustained implementation, outcomes and scaling up [8]. It follows that when interventions are evaluated in terms of sustainability, the focus is often in relation to the intervention itself, with a disconnect

For peer review only - http://bmjopen.bmj.com/site/about/guidelines.xhtml

between the intervention being sustained and its contribution to a sustainable health system. This limitation is evident in the measurement of sustainability, which is often undertaken in in terms of continuity of program activities and outcomes, institutionalisation, adaptation of the intervention components, and sustained attention to the issue or problem [9-12]. As a result, there are a growing number of theories, models and frameworks of sustainability that address individual programs and interventions [8,13,14]. These theories, models and frameworks provide valuable guidance and insights into the facilitators and barriers that contribute to intervention sustainability. In addition they provide guidance around funding of interventions that are more likely to continue beyond the implementation period as well as guidance for planning and evaluation [9,10,13-15]. While sustainability of effective programs and interventions at the end of the research translation pipeline is valuable, not all interventions need to be sustained in order to be useful or effective [10]. The process of research translation offers a number of other potential benefits to heath system sustainability such as collaboration and capacity building, along with the impact that comes from sustained implementation.

A research gap therefore emerges, with the contribution of research translation interventions on sustainability at the macro level of the health system, not well described or understood [16]. This gap becomes particularly evident when the complex adaptive nature of health systems is acknowledged. As social systems, health systems can be viewed as complex adaptive systems characterised by dynamic, non-linear interactions amongst numerous components and across all levels of the system [14,17]. The different levels of the system are defined here based on an adaptation of Caldwell and Mays [18] and Pope, et al. [19] conceptualisation of the micro, meso and macro levels of the public health care delivery system in the UK. Here we apply this conceptualisation to the Australian health system, defining the micro frame as the intervention, the meso as an intermediary frame (e.g. organisation within which the intervention is implemented) and the macro as the centralised policy-making level enacted by the department of health. This dynamic interaction across the levels of the system means that events at one level of the system influence action and events at another level in a feedback loop relationship [20]. It follows that, in a similar fashion to Pope, et al. [19], our interest is not solely with individual interventions but rather with the interconnections, interdependencies and feedback loops across a large complex system. In this context, sustainability is often considered only at the micro level [15].

As previously mentioned, sustained research translation interventions are valuable to the macro system in terms of sustaining health outcomes, an intervention that is not sustained may also contribute to health system sustainability. The value of such interventions may not come through health outcomes, but other related outputs such as collaboration, skill development and capacity

For peer review only - http://bmjopen.bmj.com/site/about/guidelines.xhtml

building that maintain the health systems capacity to deliver health over time [10,21]. These, non-health related outputs are thought to be associated with the process of undertaking research translation, and may include, interprofessional collaboration, workforce capacity building and improved value consciousness amongst clinicians [2,10]

Whilst research regarding the key factors that influence the sustainability of research translation interventions is important, the next stage is to move beyond the intervention itself, and investigate how the process of research translation can trigger change within the wider health system [14,16,22]. This requires a shift away from linear cause and effect evaluation design to methods that are capable of dealing with complexity [23]. The process of research translation can be understood as a disruption to the system which is likely to trigger emergent, adaptive behaviours and nonlinear, feedback interactions across the system which will either reinforce or diminish overall system sustainability [23]. It follows that to understand sustainability at the macro level of the system, researchers need to shift their focus from *what* has been achieved, to *how* the process generates change in the system [24]. The concept of *how* and *why* change occurs within a system is core to the theory driven evaluation design, realist evaluation [24-27]

Realist evaluation

Realist evaluation is a theory driven evaluation approach grounded in realism, a philosophy which asserts that both the material and social worlds are real [25,27]. It follows that the reality of the social and material world should be investigated to build a better understanding of what causes change within such systems. Realist evaluation shifts the basic inquiry of evaluation from “does this work?”, to “what works, in what context and how?” [25]. The realist evaluation approach can be said to be in contrast to traditional linear cause and effect approaches where complexity is controlled for or eliminated [28]. Instead, realist evaluation embraces complexity by acknowledging that context can influence the way an intervention achieves its outcomes through different change mechanisms that are triggered by context. Therefore, the realist approach aims to link intervention to outcome by identifying the various change mechanisms taking place in reality [25]. Within the realist evaluation framework, interventions are ‘theories incarnate’ embedded in a social reality that makes them prone to interpretation and modification [29,30]. Often these theories are not explicit and exist in the minds of those who designed a particular intervention [25]. It is therefore a central task of realist evaluation to make the theories explicit in the form of a program theory.

Program theories, in their simplest form, depict how a specific intervention is theorized to achieve change [31]. The realist evaluation frameworks seek to develop program theories as Context-Mechanism-Outcome (CMO) configurations that explain how interventions trigger different change

For peer review only - http://bmjopen.bmj.com/site/about/guidelines.xhtml

mechanisms across different contexts to achieve (or not achieve) outcomes. The theory is then tested and refined through data collection and analysis that assesses patterns and regularities not only in the program outputs but also, the program context, the process of implementation, and the mechanisms that may be creating change [25,26]. The outputs from the realist approach lead to a well-articulated program theory as well as a more generalizable theory, often termed a middle range theory, falling between program and grand theory that can be applied across different settings [31]. In the context of health system sustainability, the interest is in the process by which interventions trigger the change process across all levels of the system, from micro intervention level through to the macro policy level of the system. Many parallels can be drawn between the principles underlying realist evaluation and that of systems theory and complexity theory. When adding a system dynamics lens to the evaluation, it becomes clear that the CMO configurations that are central to realist evaluation involve complex, non-linear interactions often in the form of feedback loops. Within complex adaptive systems, the context, mechanism and outcomes are interconnected and, as such, the triggering of one mechanism can impact on the context of another [32]. Realist evaluation is said to be 'methods neutral' and thus provides a perspective and conceptual framework for evaluation rather than strict practical guidelines. [24]. This study therefore aims to apply the realist evaluation framework, drawing on system dynamics methods and principles, to explain how research interventions funded to deliver service improvement and value-based health care, impact on sustainability across all levels of a complex adaptive system.

STUDY AIMS AND OBJECTIVES

Aim:

Produce a program theory to explain how the research translation process contributes to health system sustainability through the implementation of interventions funded to deliver service improvement and value-based health care.

Objectives:

1. Identify the change mechanisms triggered by the implementation of research translation interventions that contribute to health system sustainability
2. Conduct case study analysis to explore the interconnected and feedback relationships between change mechanisms identified from objective 1, and
3. Refine the initial program theory of the impact of the research translation process on sustainability based on outcomes from objectives 1 and 2, through validation with key

For peer review only - http://bmjopen.bmj.com/site/about/guidelines.xhtml

stakeholders to inform a generalizable theory of mechanisms that influence sustainability at a health system level.

METHODS AND ANALYSIS

A realist evaluation approach is applied to this study [27]. The study draws on a number of theoretical frameworks including complexity science, complex adaptive systems theory and implementation science[23]. It adopts a realist evaluation methodology to examine the pathways by which the RTP program as a research translation process contributes to health system sustainability. Realist evaluation is increasingly used in health services research to evaluate complex health system interventions [28,33]. This approach has been chosen for its ability to cope with complexity and its focus on understanding how and why complex health interventions trigger change within complex systems. The RAMESES II reporting standards for realist evaluation will be used to structure the reporting of the study methods and analysis [28].

This study will be implemented in three phases:

1. Initial program theory development and evidence review: Development and refinement of an initial program theory for the research translation process and the impact on health system sustainability using peer reviewed literature, key informant interviews and program documentation.

2. Program theory testing: Test the initial program theory by applying it to selected case studies of interventions funded under the RTP program using document analysis, key stakeholder interviews and concept mapping workshops with key stakeholders.

3. Theory consolidation: Refine and validate the initial program theory based on the study findings and, peer-reviewed literature where relevant and focus group discussions with relevant stakeholders including program managers, existing and past investigators of funded projects, and members of the RTP selection panel.

The Research Translation Projects (RTP) Program

The RTP program, established in 2007 by the WA Department of Health, provides funding for short-term research projects that seek to improve healthcare practice and/or policy in the WA health system. Its aim is to improve or maintain patient outcomes through implementation of research translation projects that have the potential to deliver efficiencies to the WA health system. In an environment of rising healthcare burden and limited resources, the RTP program supports innovation with a focus on high-value care and needs-led research, funding work that might

For peer review only - http://bmjopen.bmj.com/site/about/guidelines.xhtml

otherwise not have been funded and also providing a focus on issues of importance as identified from the bottom up. It had been recognised that clinicians were generally uncompetitive for nationally funded grant programs due to lack of research training and research track-record. However, they were ideally placed to identify possible opportunities to implement new practices that promote efficiency while maintaining or improving patient outcomes. The RTP program was therefore implemented to provide an opportunity to deliver bottom up innovation and to build research capacity within the clinical environment through enablement and collaboration with those who have experience in research.

The program is innovative in terms of the requirement for inclusion of health resource appraisal, with a funding criterion that the proposed intervention would reduce or maintain costs to improve health outcomes (or reduce costs and maintain health outcomes). The combination of bottom up clinician led research translation, based on front line need and a key focus on health system efficiency, encourages clinicians from the WA health system to undertake research that has the potential to improve the quality and efficiency of their work. Through research translation into practice, innovation based on frontline need, development of research capacity within the clinical environment, collaboration amongst research and clinical workforce and the promotion of value-consciousness amongst clinicians, the RTP program also aims to contribute to the sustainability of the WA health system. Research translation projects sit along the research translation continuum and are made up of new research, pilot-studies or applications and evaluations of research that has been applied elsewhere.

Given the spread of projects funded by the RTP program, across multiple settings and addressing a variety of health and health service delivery issues, the program can be viewed as a microcosm of the broader health system, given a shared focus on making health services more effective and efficient. The RTP program therefore provides a unique opportunity to study how the process of research translation through clinician led research can trigger change mechanisms to produce effects that may contribute to sustainability from the micro to the macro levels of the system.

Phase 1: Initial program theory development and evidence review

A program theory describes how the intervention is expected to bring about changes. The initial program theory is essential to the realist evaluation logic of inquiry [27]. Developing a program theory shifts the understanding of how an intervention is expected to work from the implicit ideas that often exist in the heads of policy makers, decision makers, clinicians and researchers, to the explicit. According to Luck (2017), a program theory consists of both a theory of action and a theory of change. Program theories in terms of the theory of action and expected outcomes can often be

For peer review only - http://bmjopen.bmj.com/site/about/guidelines.xhtml

identified in policy or program documents. However in the context of a realist evaluation further information about the theory of change, that is the mechanism by which the action will achieve the outcomes, is not so obvious and requires the researcher to conduct focused interviews with key stakeholders to elicit the theory of change [34].

Data sources: The development of the program theory will be informed by three main sources: input from the key stakeholders of the RTP program, analysis of documentation and a rapid review of the literature [35]. Program stakeholders such as program managers, existing and past investigators of funded projects, and members of the RTP selection panel will be interviewed to elicit the hypothesised change mechanisms that explain how the research translation process is thought to influence health system sustainability. In addition, program documents, including RTP research applications, progress reports and final reports of funded projects as well as past evaluations of the program will be accessed. Finally a rapid review of the literature will be undertaken to identify published theories of change (e.g. theories focusing on change within the individual professionals, within the organisation, the social setting or economic context) that provide insight into effective implementation of change through the research translation process [36].

Data analysis*:* The CMO framework will underpin an iterative process of reflection and adaptation, which will take place to analyse data and identify relations between contexts, mechanisms of change and outcomes. The emergent behaviour of complex adaptive systems such as the health system would suggest that feedback loops would exist between CMO; therefore, the IPT will be depicted using a causal loop diagram (CLD) in order to capture any feedback interactions between CMOs. While logic models are commonly used to depict a program theory, the complexity of both the intervention and the system in which it is being implemented lends itself to system dynamics methods to depict the program theory.

Phase 2: Program theory testing

We will test the program theory of the research translation process developed in phase one by analysing in detail a series of RTP program case studies. Realist evaluation aims to test program theories for the purpose of refining them as well as informing implementation of the program. As such the core questions asked of the program are; what works (and does not work) for whom, in what circumstances and how? Mechanisms that produce positive outcomes in one context may not produce the same outcomes in an alternative context. This notion that the success (or failure) of an intervention is context dependant is one shared in the current literature that explores implementation science and complex interventions (Braithwaite, Churruca, Long, Ellis, & Herkes, 2018). Anderson and Hardwick (2016) outline this concept succinctly:

For peer review only - http://bmjopen.bmj.com/site/about/guidelines.xhtml

"Recognising that no one policy, programme or intervention will always work, all the time, for everyone, realist approaches seek to explain this pattern of outcomes through building programme theories about how an intervention (policy or programme) is meant to work (often according to programme architects, or policymakers, or participants), and then 'test' whether and how this programme theory plays out in the real world using empirical data." (Anderson & Hardwick, 2016, p. 325)

Selection criteria*:* Given the variety of implemented research projects funded by the RTP program, a descriptive analysis of past RTP projects will be undertaken prior to a case study selection to enable a selection of cases that captures the variety of activity undertaken within the RTP program. Projects will be classified based on characteristics such as context of implementation (e.g. ,metro hospital, rural hospital, primary care, community health) and type of research translation intervention (e.g. new practice guideline, new service, new test, role substitution) [8]. In the first instance, case study selection will be purposive in nature to ensure a breadth of projects across the above criteria is captured. However, given the retrospective nature of this study, case study selection may be limited by access to project investigators and other stakeholders for interview.

Data sources: Case study analysis will be undertaken using three data collection techniques: key informant interviews, document review and analysis of individual RTP reports and other key documents, and secondary data analysis of project data such as cost and outcome data where necessary.

Data analysis*:* Thematic analysis will involve a process of organising data in relation to the initial programme theory (phase 1), whereby the data is mapped against the CLD developed in phase 1. Additionally, data will be coded and themed according to patterns of context, mechanism and outcomes. This thematic analysis will be informed by the Consolidated Framework for Implementation Research (CFIR), a widely used implementation science framework providing a typology to promote implementation theory development about what works, where and why across multiple settings [37]. The aim of this phase in the realist approach is to identify emerging patterns across the case studies to validate and refine the initial program theory to be consolidated in phase three. In addition, the interactions and relationships between change mechanisms will be refined.

Phase 3: Theory consolidation

The final phase of research will involve refining the initial program theory to produce a final iteration of the research translation process to be presented in the form of a mid-range program theory of its impact on sustainability across the micro, meso and macro levels of the system. The final model will

For peer review only - http://bmjopen.bmj.com/site/about/guidelines.xhtml

articulate a robust and empirically tested model of complex relationships among program and health service unit, organisation and policy level outcomes for sustainability. Similar to the process undertaken in phase one, the theory consolidation will be informed through the input of key stakeholders of the RTP program and a rapid review of the literature to update the initial rapid review to include relationships and interconnections between change mechanisms. Input from key stakeholders will be elicited through concept mapping workshops and interviews where the theory will be presented to key stakeholders for validation.

Implementation and evaluation recommendations for RTP program*:* In addition to the refined RTP theory, a Delphi Survey of experts will be undertaken to determine implementation and evaluation recommendations for the RTP program in the future. The survey will be conducted across a number of rounds via online questionnaire, which will be delivered until consensus is reached. The Delphi panel will consist of those involved in delivering the RTP program at the WA Department of Health, investigators involved in individual projects and other stakeholders. These recommendations will include an evaluation framework specifically for the RTP program to be addressed by future funding recipients and selection panels.

For peer review only

For peer review only - http://bmjopen.bmj.com/site/about/guidelines.xhtml

REFERENCES

1. Braithwaite J, Zurynski Y, Ludlow K, et al. Towards sustainable healthcare system performance in the 21st century in high-income countries: a protocol for a systematic review of the grey literature. *BMJ Open* 2019;9(1) doi: 10.1136/bmjopen-2018-025892
2. Crisp N. What would a sustainable health and care system look like? *BMJ* 2017;358 doi: 10.1136/bmj.j3895
3. Sustainable Health Review. Sustainable Health Review: Final Report to the Western Australian Government. Western Australia.: Department of Health, 2019.
4. Braithwaite J, Testa L, Lamprell G, et al. Built to last? The sustainability of health system improvements, interventions and change strategies: a study protocol for a systematic review. *BMJ open* 2017;7(11):e018568-e68. doi: 10.1136/bmjopen-2017-018568
5. Cruz Rivera S, Kyte DG, Aiyegbusi OL, et al. Assessing the impact of healthcare research: A systematic review of methodological frameworks. *PLOS Medicine* 2017;14(8):e1002370. doi: 10.1371/journal.pmed.1002370
6. Wensing M, Grol R. Knowledge translation in health: how implementation science could contribute more. *BMC medicine* 2019;17(1):88. doi: 10.1186/s12916-019-1322-9
7. Government of Western Australia; Department of Health. Research Translation Projects 2019 [Available from: https://ww2.health.wa.gov.au/Articles/N_R/Research-Translation-Projects.
8. Tricco AC, Ashoor HM, Cardoso R, et al. Sustainability of knowledge translation interventions in healthcare decision-making: a scoping review. *Implementation science : IS* 2016;11(55):55-55. doi: 10.1186/s13012-016-0421-7
9. Shelton RC, Cooper BR, Stirman SW. The Sustainability of Evidence-Based Interventions and Practices in Public Health and Health Care. *Annu Rev Public Health* 2018;39(1):55-76. doi: 10.1146/annurev-publhealth-040617-014731
10. Whelan J, Love P, Pettman T, et al. Cochrane Update: Predicting sustainability of intervention effects in public health evidence: identifying key elements to provide guidance. *Journal of Public Health* 2014;36(2):347-51. doi: 10.1093/pubmed/fdu027
11. Lennox L, Maher L, Reed J. Navigating the sustainability landscape: a systematic review of sustainability approaches in healthcare. *Implement Sci* 2018;13(1) doi: 10.1186/s13012-017-0707-4
12. Flynn R, Scott SD. Understanding Determinants of Sustainability Through a Realist Investigation of a Large-Scale Quality Improvement Initiative (Lean): A Refined Program Theory. *Journal of nursing scholarship : an official publication of Sigma Theta Tau International Honor Society of Nursing* 2019 doi: 10.1111/jnu.12527
13. Stirman S, Kimberly J, Cook N, et al. The sustainability of new programs and innovations: a review of the empirical literature and recommendations for future research. *Implement Sci* 2012;7(1) doi: 10.1186/1748-5908-7-17
14. Gruen RL, Elliott JH, Nolan ML, et al. Sustainability science: an integrated approach for health-programme planning. *The Lancet* 2008;372(9649):1579-89. doi: 10.1016/S0140-6736(08)61659-1
15. Braithwaite J, Ludlow K, Testa L, et al. Built to last? The sustainability of healthcare system improvements, programmes and interventions: a systematic integrative review. *BMJ open* 2020;10(6):e036453. doi: 10.1136/bmjopen-2019-036453
16. Greenhalgh T, Macfarlane F, Barton-Sweeney C, et al. "If We Build It, Will It Stay?" A Case Study of the Sustainability of Whole-System Change in London. *Milbank Quarterly* 2012;90(3):516-47. doi: 10.1111/j.1468-0009.2012.00673.x
17. Hawe P, Shiell A, Riley T. Theorising Interventions as Events in Systems. *American Journal of Community Psychology* 2009;43(3-4):267-76. doi: 10.1007/s10464-009-9229-9
18. Caldwell SEM, Mays N. Studying policy implementation using a macro, meso and micro frame analysis: the case of the Collaboration for Leadership in Applied Health Research & Care

For peer review only - http://bmjopen.bmj.com/site/about/guidelines.xhtml

(CLAHRC) programme nationally and in North West London. *Health research policy and systems* 2012;10:32-32. doi: 10.1186/1478-4505-10-32
19. Pope C, Robert G, Bate P, et al. Lost in translation: A multi-level case study of the metamorphosis of meanings and action in public sector organizational innovation. *Public Adm* 2006;84(1):59-79.
20. Innovative Care for Chronic Conditions : Building Blocks for Action. Geneva: Geneva : World Health Organization 2002.
21. Chambers DA, Glasgow RE, Stange KC. The dynamic sustainability framework: addressing the paradox of sustainment amid ongoing change. *Implementation science : IS* 2013;8(1):117-17. doi: 10.1186/1748-5908-8-117
22. Hedelin B. Complexity is no excuse. *Sustainability Science* 2019;14(3):733-49. doi: 10.1007/s11625-018-0635-5
23. Braithwaite J, Churruca K, Long JC, et al. When complexity science meets implementation science: a theoretical and empirical analysis of systems change. *BMC medicine* 2018;16(1):63. doi: 10.1186/s12916-018-1057-z
24. Wong G, Greenhalgh T, Westhorp G, et al. Realist methods in medical education research: what are they and what can they contribute? *Medical Education* 2012;46(1):89-96. doi: 10.1111/j.1365-2923.2011.04045.x
25. Pawson R. The science of evaluation : a realist manifesto / Ray Pawson. London: London : SAGE 2013.
26. Greenhalgh T, Wong G, Jagosh J, et al. Protocol—the RAMESES II study: developing guidance and reporting standards for realist evaluation. *BMJ Open* 2015;5(8) doi: 10.1136/bmjopen-2015-008567
27. Pawson R. Realistic evaluation / Ray Pawson and Nick Tilley. London: London : SAGE 1997.
28. Geoff W, Gill W, Joanne G, et al. Quality and reporting standards, resources, training materials and information for realist evaluation: the RAMESES II project. *Health Services and Delivery Research* 2017;5(28) doi: 10.3310/hsdr05280
29. Rycroft-Malone J, Burton C, Wilkinson J, et al. Collective action for knowledge mobilisation: a realist evaluation of the Collaborations for Leadership in Applied Health Research and Care. *Health Services and Delivery Research* 2015;3(44) doi: 10.3310/hsdr03440
30. Nurjono M, Shrestha P, Lee A, et al. Realist evaluation of a complex integrated care programme: protocol for a mixed methods study. *BMJ Open* 2018;8(3) doi: 10.1136/bmjopen-2017-017111
31. Shearn K, Allmark P, Piercy H, et al. Building Realist Program Theory for Large Complex and Messy Interventions. *International Journal of Qualitative Methods* 2017;16(1) doi: 10.1177/1609406917741796
32. Plsek PE, Greenhalgh T. The challenge of complexity in health care. *BMJ* 2001;323(7313):625. doi: 10.1136/bmj.323.7313.625
33. Moore GF, Audrey S, Barker M, et al. Process evaluation of complex interventions: Medical Research Council guidance. *BMJ : British Medical Journal* 2015;350(mar19 6) doi: 10.1136/bmj.h1258
34. Luck K. A proposed program theory for a smoke-free hospital property. *Soc Theory Health* 2017;15(3):323-45. doi: 10.1057/s41285-017-0031-8
35. Manzano A. The craft of interviewing in realist evaluation. *Evaluation* 2016;22(3):342-60. doi: 10.1177/1356389016638615
36. Grol R, Wensing M, Eccles M, et al. Improving patient care: the implementation of change in health care2013.
37. Damschroder LJ, Aron DC, Keith RE, et al. Fostering implementation of health services research findings into practice: a consolidated framework for advancing implementation science. *Implementation science : IS* 2009;4(1):50. doi: 10.1186/1748-5908-4-50

For peer review only - http://bmjopen.bmj.com/site/about/guidelines.xhtml

For peer review only

Authors ‘contribution: AM, DH, EG, YZ, SR jointly conceived the study; AM and DH developed the study proposal; AM led the writing of this paper; DH, EG, YZ and SR read and reviewed the final version prior to submission.

Funding statement: This work is supported by the National Health and Medical Research Council Partnership Centre for Health System Sustainability (ID: 9100002). In addition the Western Australian Department of Health provides financial support for the Research Translation Projects program.

Competing interests: None declared.

Patient consent: Not required.

Ethics approval: Curtin University Human Research Ethics Committee (HREC) has approved this study. Approval number: HRE2020-0464

Provenance and peer review: Not commissioned; peer reviewed for ethical and funding approval prior to submission.

PPI statement: Patients and the Public were not involved in the design of this protocol. However public participation does occur in the research translation projects program and will also be involved in conducting the research outlined in this protocol paper.

Word count: 3,874 words.

For peer review only - http://bmjopen.bmj.com/site/about/guidelines.xhtml

BMJ Open

Realist evaluation of the impact of the research translation process on health system sustainability: a study protocol

Journal:	*BMJ Open*
Manuscript ID	bmjopen-2020-045172.R1
Article Type:	Protocol
Date Submitted by the Author:	04-Jun-2021
Complete List of Authors:	Mosedale, Abby; Curtin University, School of Public Health<br>Hendrie, Delia; Curtin University, School of Public Health<br>Geelhoed, Elizabeth; Telethon Kids Institute<br>Zurynski, Yvonne; Macquarie University, Australian Institute of Health Innnovation<br>Robinson, Suzanne; Curtin University, Health Policy Management
**Primary Subject Heading**:	Health services research
Secondary Subject Heading:	Evidence based practice, Health policy
Keywords:	Organisation of health services < HEALTH SERVICES ADMINISTRATION & MANAGEMENT, PUBLIC HEALTH, Quality in health care < HEALTH SERVICES ADMINISTRATION & MANAGEMENT

SCHOLARONE™
Manuscripts

I, the Submitting Author has the right to grant and does grant on behalf of all authors of the Work (as defined in the below author licence), an exclusive licence and/or a non-exclusive licence for contributions from authors who are: i) UK Crown employees; ii) where BMJ has agreed a CC-BY licence shall apply, and/or iii) in accordance with the terms applicable for US Federal Government officers or employees acting as part of their official duties; on a worldwide, perpetual, irrevocable, royalty-free basis to BMJ Publishing Group Ltd ("BMJ") its licensees and where the relevant Journal is co-owned by BMJ to the co-owners of the Journal, to publish the Work in this journal and any other BMJ products and to exploit all rights, as set out in our licence.

The Submitting Author accepts and understands that any supply made under these terms is made by BMJ to the Submitting Author unless you are acting as an employee on behalf of your employer or a postgraduate student of an affiliated institution which is paying any applicable article publishing charge ("APC") for Open Access articles. Where the Submitting Author wishes to make the Work available on an Open Access basis (and intends to pay the relevant APC), the terms of reuse of such Open Access shall be governed by a Creative Commons licence – details of these licences and which Creative Commons licence will apply to this Work are set out in our licence referred to above.

Other than as permitted in any relevant BMJ Author's Self Archiving Policies, I confirm this Work has not been accepted for publication elsewhere, is not being considered for publication elsewhere and does not duplicate material already published. I confirm all authors consent to publication of this Work and authorise the granting of this licence.

For peer review only

TITLE

Realist evaluation of the impact of the research translation process on health system sustainability: a study protocol

Corresponding author: *Ms Abby Mosedale*. School of Public Health, Curtin University, Perth, Australia.

Email: abby.mosedale@curtin.edu.au

Address: Kent Street, Bentley, Perth

Western Australia, 6102

Tel: +61 8 9266 9266 Fax: +61 8 9266 2255

Co-authors:

Dr Delia Hendrie.

School of Public Health, Curtin University, Perth, Australia.

Professor Elizabeth Geelhoed.

Telethon Kids Institute, Perth, Australia

Associate Professor Yvonne Zurynski

Australian Institute of Health Innovation, Macquarie University, Sydney, Australia

NHMRC Partnership Centre for Health System Sustainability, Macquarie University, Sydney, Australia

Professor Suzanne Robinson.

School of Public Health, Curtin University, Perth, Australia.

Funding statement: This work is supported by the National Health and Medical Research Council Partnership Centre for Health System Sustainability (ID: 9100002). In addition the Western Australian Department of Health provides financial support for the Research Translation Projects program.

Word count: 4158 words

For peer review only

For peer review only - http://bmjopen.bmj.com/site/about/guidelines.xhtml

ABSTRACT

Introduction: Sustainability at a system level relates to the capacity of the system to be able to service the ongoing health needs of the population. It is a multifaceted concept encompassing both the affordability and efficiency of a system and the system's ability to adapt and change.

To address issues that currently threaten health system sustainability, health care leaders, policy makers, clinicians and researchers are searching for solutions to ensure the delivery of safe, value-based care into the future. The timely translation of research evidence into sustainable interventions that can be adopted into the health system is one way of bolstering the sustainability of the system as a whole. We present a protocol for the realist evaluation of a research translation funding program to understand how the research translation process contributes to health system sustainability and value-based health care.

Methods and analysis:

Underpinned by the realist evaluation framework, we will: (1) develop the Initial Program Theory (IPT) of the research translation process; (2) test the program theory through case study analysis; and (3) refine and consolidate the theory through stakeholder consultation. The evaluation uses a case example of a research translation program, chosen for its representation of a microcosm of the broader health system and the heterogeneity of service improvement activities taking place within it. Across the three phases, analysis of data from documents about the research translation program and interviews and focus groups with stakeholders and program users will draw on the context (C), mechanism (M), outcome (O) formula that is core to realist evaluation. In addition, system dynamic methods will capture the feedback loops and complex relationships amongst the IPT and CMO configurations. This approach to evaluation of a research translation funding program may be adapted to similar programs operating in other settings.

STRENGTHS AND LIMITATIONS OF THIS STUDY

- This is the first study to evaluate the impact of the research translation process on health system sustainability.
- Adopting a realist methodology will allow contextual factors and mechanisms to be identified that influence how the research translation process can contribute to health system sustainability.

For peer review only - http://bmjopen.bmj.com/site/about/guidelines.xhtml

- A novel feature of this study is the application of system dynamics modelling within the realist evaluation framework, capturing the complexity and feedback relationships between context, mechanism and outcome.
- Using a Delphi method to establish consensus on implementation and evaluation recommendations for a research translation program will facilitate perspectives to be gained from a broad group of stakeholders.
- Evaluation of one research translation program as a case example may affect generalisability of study findings to the research translation process more generally.

INTRODUCTION

Health systems around the world are facing challenges relating to rising healthcare expenditure and the effectiveness and efficiency of the system to deliver high value[1], safe healthcare. A number of complex interdependent factors contribute to these challenges. An aging population, rising chronic disease, public expectations, and a lack of value-consciousness amongst healthcare consumers and providers are the major factors driving growing demand for healthcare [2,3]. These issues are occurring in an environment that is resistant to change, often driven by behaviours associated with vested interests, and incentives that do not promote value or transparency [4]. These challenges, pressures and behaviours raise questions about capacity of the system to deliver affordable, cost-effective outcomes to the population over time, which is often referred to as the sustainability of the system [5]. Research translation, is an essential process for ensuring health systems have ongoing capacity to service the health needs of a population and address challenges through the integration of cost-effective interventions based on new research and technology. While there is a wide range of definitions in the literature in relation to research translation and other associated terminology such as knowledge translation, and research utilisation[6], a working definition of research translation for the purposes of this study has been adopted from Grimshaw and colleagues, as "ensuring that stakeholders are aware of and use research evidence to inform their health and healthcare decision making"[7].

Western Australia (WA) is typical of other jurisdictions in Australia facing challenges that threaten the long-term sustainability of its health system. The demand for health services has grown substantially in recent years, along with health expenditure, yet outcomes in population health and acute care in WA have not improved at the same rate [4]. With major health issues such as increasing obesity, an aging population, chronic disease, mental health and inequalities in health outcomes across the WA population, the sustainability of the WA health system is a growing concern for healthcare leaders and policy makers. The WA Department of Health, along with healthcare leaders,

For peer review only - http://bmjopen.bmj.com/site/about/guidelines.xhtml

policy makers, clinicians and researchers around the globe, acknowledge the potential of research evidence to improve health outcomes and optimise resource use [4,8,9]. In 2007 the former State Health Research Advisory Council within the WA Department of Health, introduced the Research Translation Projects (RTP) program [10]. The RTP program aims to cultivate and translate evidence for a sustainable health system through the support of health service driven, high-quality research projects that have the potential to deliver improved cost effectiveness and/or efficiencies to the health system while maintaining or improving patient outcomes. Internal evaluations of the RTP program have focused primarily on research outputs such as the number of publications, changes to practice guidelines and additional research funding obtained. Whilst assessment of research outputs is important, the RTP program's contribution to health system sustainability is not well understood. Therefore, the protocol presented here aims to outline the theories and methods to explain how the RTP program supports research translation that in turn contributes to health system sustainability. We provide a rationale for adopting a realist evaluation framework to underpin the evaluation of the RTP program with the aim of contributing to a more generalizable understanding of the relationships between the process of research translation and its contribution to health system sustainability.

BACKGROUND AND RATIONALE

Sustainability from a health systems perspective relates to the capacity of the system to be able to service the health needs of the population into the future[5]. It is a multifaceted concept that encompasses both the affordability and efficiency of a system, and the system's ability to adapt and change [3]. Adopting a system thinking approach gives us a framework for understanding the health system as entities, made up of sub-systems such as interventions, programs, organisations, stakeholders and agencies (e.g. the emergency departments, quality improvement programs, safety policies, the workforce, mangers and decision-makers and consumers). In this protocol "health system" means the broader entity as described above. This is an important assertion to make considering the wide-ranging use of the term 'health system sustainability" used in the literature. For example, the term health system sustainability is often used in literature in relation to the discrete sub-systems described above (e.g. intervention, organisation and agencies). The sustainability of the broader health system, which is the focus here, is not well understood and the question of how does action in the form of multiple research translation activities and projects impact on the sustainability of the wider health system remains unanswered. To address issues that currently threaten health system sustainability, healthcare leaders, policy makers, clinicians and researchers are searching for solutions that capitalise on new research and technologies that

For peer review only - http://bmjopen.bmj.com/site/about/guidelines.xhtml

promise to deliver safer, value-based care. Such solutions often involve implementing and testing new evidence-based interventions and improvement programs into the health system, and evaluating their effectiveness, often through pragmatic trials [11,12].

Increasingly, evaluations also include a focus on the sustainability of the interventions, involving recognition of implementation outcomes such as acceptability, adoption into policy or practice, appropriateness, reach and sustainability to support the spread and adaptation of successful interventions beyond the original setting and context [11].

As a result, there are a growing number of theories, models and frameworks of sustainability that address individual programs and interventions [11,13,14]. These theories, models and frameworks provide valuable guidance and insights into the facilitators and barriers that contribute to intervention sustainability [15]. In addition, they provide guidance around funding of interventions that are more likely to continue providing desirable benefits beyond the implementation period usually covered by project funding, as well as guidance for planning and evaluation [13,14,16-18]. The contribution of health improvement interventions to sustainability at the broader system level has received limited attention. This limitation is evident in the measurement of sustainability presented in the literature, which is often presented in terms of continuity of program activities and outcomes, institutionalisation, adaptation of the intervention components, and sustained attention to the issue or problem [16,17,19,20].

While sustainability of effective programs and interventions at the end of the research translation pipeline is valuable, not all interventions need to be sustained in order to be useful or to make a contribution to the larger system goals in which they operate[17]. The process and strategies adopted to facilitate research translation offers several other potential benefits to heath system sustainability such as workforce collaboration and capacity building, along with the impact that comes from successful implementation[17,21].

The understanding of research translation as a process within the broader health system, and its contribution to system sustainability needs further exploration. In view of health systems as complex adaptive systems [14,22] the dynamic interaction between and within the system and subsystems means that events at one level of the system influence action and events at another, often in a feedback loop relationship [23]. It follows that, our interest is not solely with individual interventions but rather with the interconnections, interdependencies and feedback loops across a large complex system. Whilst research regarding the key factors that influence the sustainability of interventions is important, the next stage is to move beyond the intervention itself, and investigate how the process of research translation can trigger change within the wider health system [14,24,25]. This requires a shift

For peer review only - http://bmjopen.bmj.com/site/about/guidelines.xhtml

away from linear cause and effect evaluation design to methods that are capable of dealing with complexity [26]. The process of research translation can be understood as a disruption to the system which is likely to trigger emergent, adaptive behaviours and nonlinear, feedback interactions across the system which will either reinforce or diminish overall system sustainability [26]. Therefore, to understand sustainability at the health system level, researchers need to shift their focus from *what* has been achieved, to *how* the process generates change in the system [27] to Identify system traps and opportunities that produce system behaviour. The concept of *how* and *why* change occurs within a system is core to the theory driven evaluation design, realist evaluation [27-30].

Realist evaluation

Realist evaluation is a theory driven evaluation approach grounded in realism, a philosophy which asserts that both the material and social worlds are real [28,30]. It follows that the reality of the social and material world should be investigated to build a better understanding of what causes change within such systems. Realist evaluation shifts the basic inquiry of evaluation from "does this work?", to "what works, in what context and how?" [28]. The realist evaluation approach can be said to be in contrast to traditional linear cause and effect approaches where complexity is controlled for or eliminated [31]. Instead, realist evaluation embraces complexity by acknowledging that context can influence the way an intervention achieves its outcomes through different change mechanisms that are triggered by context. Therefore, the realist approach aims to link intervention to outcome by identifying the various change mechanisms taking place in reality [28]. Within the realist evaluation framework, interventions are 'theories incarnate' embedded in a social reality that makes them prone to interpretation and modification [32,33]. Often these theories are not explicit and exist in the minds of those who designed a particular intervention [28]. It is therefore a central task of realist evaluation to make the theories explicit in the form of a program theory.

Program theories, in their simplest form, depict how a specific intervention is theorized to achieve change [34]. The realist evaluation frameworks seek to develop program theories as Context-Mechanism-Outcome (CMO) configurations that explain how interventions trigger different change mechanisms across different contexts to achieve (or not achieve) outcomes. The theory is then tested and refined through data collection and analysis that assesses patterns and regularities not only in the program outputs but also the program context, the process of implementation, and the mechanisms that may be creating change [28,29]. The outputs from the realist approach lead to a well-articulated program theory as well as a more generalizable theory, often termed a middle range theory, falling between program and grand theory that can be applied across different settings [34]. In the context of health system sustainability, the interest is in the process by which interventions

For peer review only - http://bmjopen.bmj.com/site/about/guidelines.xhtml

trigger the change process across all levels of the system, from micro intervention level through to the macro policy level of the system. Many parallels can be drawn between the principles underlying realist evaluation and that of systems theory and complexity theory. When adding a system dynamics lens to the evaluation, it becomes clear that the CMO configurations that are central to realist evaluation involve complex, non-linear interactions often in the form of feedback loops. Within complex adaptive systems, the context, mechanism and outcomes are interconnected and, as such, the triggering of one mechanism can impact on the context of another [35]. Realist evaluation is said to be 'methods neutral' and thus provides a perspective and conceptual framework for evaluation rather than strict practical guidelines. [27]. This study therefore aims to apply the realist evaluation framework, drawing on system dynamics methods and principles, to explain how research interventions, funded to deliver service improvement and value-based health care, impact on sustainability across all levels of a complex adaptive system.

STUDY AIMS AND OBJECTIVES

Aim:

Produce a program theory to explain how the research translation process contributes to health system sustainability through the implementation of interventions funded to deliver service improvement and value-based health care.

Objectives:

1. Identify the change mechanisms triggered by the implementation of research translation interventions that contribute to health system sustainability
2. Conduct case study analysis to explore the interconnected and feedback relationships between change mechanisms identified from objective 1, and
3. Refine the initial program theory of the impact of the research translation process on health system sustainability based on outcomes from objectives 1 and 2, through validation with key stakeholders to inform a generalizable theory of mechanisms that influence sustainability at a health system level.

For peer review only - http://bmjopen.bmj.com/site/about/guidelines.xhtml

METHODS AND ANALYSIS

A realist evaluation approach is applied to this study [30]. The study draws on a number of theoretical frameworks including complexity science, complex adaptive systems theory and implementation science[26]. It adopts a realist evaluation methodology to examine the pathways by which the RTP program as a research translation process contributes to health system sustainability. Realist evaluation is increasingly used in health services research to evaluate complex health system interventions [31,36]. This approach has been chosen for its ability to cope with complexity and its focus on understanding how and why complex health interventions trigger change within complex systems. The RAMESES II reporting standards for realist evaluation will be used to structure the reporting of the study methods and analysis [31]. To enhance the trustworthiness of data collection and documentation throughout the project, we will follow Pawson, et al. [37] Transparency, Accuracy, Purposivity, Utility, Propriety, Accessibility (TAPUPAS) quality standards framework [38]. Patients and the public were not involved in the design of this study. However public participation does occur in the research translation projects program and will also be involved in conducting the research outlined in this protocol paper.

This study will be implemented in three phases, summarised in Table 1

1. Initial program theory development and evidence review: Development and refinement of an initial program theory for the research translation process and the impact on health system sustainability using peer reviewed literature, key informant interviews and program documentation.

2. Program theory testing: Test the initial program theory by applying it to selected case studies of interventions funded under the RTP program using document analysis, key stakeholder interviews and concept mapping workshops with key stakeholders.

3. Theory consolidation: Refine and validate the initial program theory based on the study findings and, peer-reviewed literature where relevant and focus group discussions with relevant stakeholders including program managers, existing and past investigators of funded projects, and members of the RTP selection panel.

For peer review only - http://bmjopen.bmj.com/site/about/guidelines.xhtml

Table 1: The three phased approach to realist evaluation of research translation in Western Australia

Phase	Data Sources	Analysis
**Phase 1: Initial program theory development and evidence review**	• Key stakeholder interviews<br>• Program documentation<br>• Peer reviewed literature	Qualitative-thematic analysis driven by the realist evaluation and system dynamics principles.
**Phase 2: Program theory testing**	• Key informant interviews<br>• Document review and analysis of individual RTP reports and other key documents<br>• Secondary data analysis of project data such as cost and outcome data (where necessary)	Iterative process of categorising and connecting strategies, similar to those proposed by Maxwell [39] will be applied to CMO development in light of the information collected during phase 2.
**Phase 3: Theory consolidation**	• Key stakeholders' interviews and workshops<br>• Peer reviewed literature<br>• Delphi Survey	Refine theory that explains how and under which contextual factors that research translation brings about health system sustainability.<br><br>Delphi survey analysis for consensus relating to recommendation for ongoing research translation practices in the Western Australian Health system.

The Research Translation Projects (RTP) Program

The RTP program, established in 2007 by the WA Department of Health, provides funding for short-term research projects that seek to improve healthcare practice and/or policy in the WA health system. Its aim is to improve or maintain patient outcomes through implementation of research translation projects that have the potential to deliver efficiencies to the WA health system. In an environment of rising healthcare burden and limited resources, the RTP program supports innovation with a focus on high-value care[1] and needs-led research, funding work that might otherwise not have been funded and also providing a focus on issues of importance as identified from the bottom up. Similar programs such as the US Department of Veteran Affairs' Diffusion of Excellence program has demonstrated positive results for implementation of evidence based practice using a bottom up approach, such as diffusion and sustainability of interventions[40]. In addition to the benefits of bottom up research, it had been recognised within the WA health system

For peer review only

For peer review only - http://bmjopen.bmj.com/site/about/guidelines.xhtml

that clinicians were generally uncompetitive for nationally funded grant programs due to lack of research training and research track-record. However, they were ideally placed to identify possible opportunities to implement new practices that promote efficiency while maintaining or improving patient outcomes. The RTP program was therefore implemented to provide an opportunity to deliver bottom up innovation and to build research capacity within the clinical environment through enablement and collaboration with those who have experience in research.

The program is innovative in terms of the requirement for inclusion of health resource appraisal, with a funding criterion that the proposed intervention would reduce or maintain costs to improve health outcomes (or reduce costs and maintain health outcomes). The combination of bottom up clinician led research translation, based on front line need and a key focus on health system efficiency, encourages clinicians from the WA health system to undertake research that has the potential to improve the quality and efficiency of their work. Through research translation into practice, innovation based on frontline need, development of research capacity within the clinical environment, collaboration amongst research and clinical workforce and the promotion of value-consciousness amongst clinicians, the RTP program also aims to contribute to the sustainability of the WA health system. Research translation projects sit along the research translation continuum and are made up of new research, pilot-studies or applications and evaluations of research that has been applied elsewhere.

Given the spread of projects funded by the RTP program, across multiple settings and addressing a variety of health and health service delivery issues, the program can be viewed as a microcosm of the broader health system, given a shared focus on making health services more effective and efficient. The RTP program therefore provides a unique opportunity to study how the process of research translation through clinician led research can trigger change mechanisms to produce effects that may contribute to sustainability across the WA health system.

Phase 1: Initial program theory development and evidence review

A program theory describes how the intervention is expected to bring about changes. The initial program theory is essential to the realist evaluation logic of inquiry [30]. Developing a program theory shifts the understanding of how an intervention is expected to work from the implicit ideas that often exist in the heads of policy makers, decision makers, clinicians and researchers, to the explicit. According to Luck (2017), a program theory consists of both a theory of action and a theory of change. Program theories in terms of the theory of action and expected outcomes can often be identified in policy or program documents. However in the context of a realist evaluation further information about the theory of change, that is the mechanism by which the action will achieve the

For peer review only - http://bmjopen.bmj.com/site/about/guidelines.xhtml

outcomes, is not so obvious and requires the researcher to conduct focused interviews with key stakeholders to elicit the theory of change [41].

Data sources: The development of the program theory will be informed by three main sources: Input from the key stakeholders of the RTP program, analysis of documentation and the literature [42]. Program stakeholders such as program managers, existing and past investigators of funded projects, and members of the RTP selection panel (clinicians, health administrators, consumer representatives, health economists) will be interviewed to elicit the hypothesised change mechanisms that explain how the research translation process is thought to influence health system sustainability. In addition, program documents, including RTP research applications, progress reports and final reports of funded projects as well as past evaluations of the program will be accessed. While the initial program theory will be informed and supported by stakeholder interviews, exploration of the literature through a theory driven review is necessary to make sense of the emerging theories elicited from interviews (i.e. hypotheses, hunches, aspirations, intuitions, experiences) ,inherent to complex interventions applied in heterogeneous contexts [43].

The literature review will follow the steps of realist synthesis review outlined by Pawson, et al. [44] and Jagosh [45] to identify published theories of change within the context of the research translation process (e.g. theories focusing on change within the individual professionals, within the organisation, the social setting or economic context) [46].

Data analysis*:* The CMO framework will underpin an iterative process of reflection and adaptation, which will take place to analyse data and identify relations between contexts, mechanisms of change and outcomes for analysis of stakeholder interviews and literature review The emergent behaviour of complex adaptive systems such as the health system would suggest that feedback loops would exist between CMO; therefore, the IPT will be depicted using a causal loop diagram (CLD) to capture any feedback interactions between CMOs. While logic models are commonly used to depict a program theory, the complexity of both the intervention and the system in which it is being implemented lends itself to system dynamics methods to depict the program theory.

Phase 2: Program theory testing and validation

We will test the program theory of the research translation process developed in phase one by analysing in detail a series of RTP program case studies. Realist evaluation aims to test program theories for the purpose of refining them as well as informing implementation of the program. As such the core questions asked of the program are; what works (and does not work) for whom, in

For peer review only - http://bmjopen.bmj.com/site/about/guidelines.xhtml

what circumstances and how? Mechanisms that produce positive outcomes in one context may not produce the same outcomes in an alternative context. This notion that the success (or failure) of an intervention is context dependent is one shared in the current literature that explores implementation science and complex interventions (Braithwaite, Churruca, Long, Ellis, & Herkes, 2018). Anderson and Hardwick (2016) outline this concept succinctly:

"Recognising that no one policy, programme or intervention will always work, all the time, for everyone, realist approaches seek to explain this pattern of outcomes through building programme theories about how an intervention (policy or programme) is meant to work (often according to programme architects, or policymakers, or participants), and then 'test' whether and how this programme theory plays out in the real world using empirical data." (Anderson & Hardwick, 2016, p. 325). The question that is at the core of the application of realist evaluation proposed here is what happens to the individual projects funded by RTP and are there common system pitfalls or opportunities that lead to their success or failure in terms of research translation.

Selection criteria***:*** Given the variety of implemented research projects funded by the RTP program, a descriptive analysis of past RTP projects will be undertaken prior to a case study selection to enable a selection of cases that captures the variety of activity undertaken within the RTP program. Projects will be classified based on characteristics such as context of implementation (e.g. metro hospital, rural hospital, primary care, community health) and type of research translation intervention (e.g. new practice guideline, new service, new test, role substitution) [11]. In the first instance, case study selection will be purposive in nature by categorising projects into a matrix of the above criteria to ensure a breadth of projects across those criteria is captured. However, given the retrospective nature of this study, case study selection may be limited by access to project investigators and other stakeholders for interview.

Data sources: Case study analysis will be undertaken using three data collection techniques: key informant interviews, document review and analysis of individual RTP reports and other key documents, and secondary data analysis of project data such as cost and outcome data where necessary.

Data analysis***:*** Qualitative data, including documentation, interviews and focus groups, will be analysed in NVivo using the CMO configuration as a guide for analysis. Several authors highlight the absence of guidance within the realist evaluation approach as to specific analytic tools to be used [47-50]. The qualitative data from the case studies will undergo an iterative process of thematic analysis by which preliminary codes will be developed for themes identified in the IPT and more specific codes and themes being induced from further cycles of thematic analysis. This will also

For peer review only - http://bmjopen.bmj.com/site/about/guidelines.xhtml

involve a process of mapping codes against the CLD developed in phase 1. The IPT will then be refined to best reflect the emerging findings. The aim of this phase in the realist approach is to identify emerging patterns across the case studies to validate and refine the initial program theory to be consolidated in phase three. In addition, the interactions and relationships between change mechanisms will be refined.

Phase 3: Theory consolidation

The final phase of research will involve refining the initial program theory to produce a final iteration of the research translation process to be presented in the form of a mid-range program theory of its impact on sustainability of the system. The final model will articulate a model of complex relationships among program and health service unit, organisation and policy level processes of research translation for sustainability of the health system. Like the process undertaken in phase one, the theory consolidation will be informed through the input of key stakeholders of the RTP program and a rapid review of the literature to update the initial realist review to include relationships and interconnections between change mechanisms. Input from key stakeholders will be elicited through concept mapping workshops and interviews where the theory will be presented to key stakeholders for validation.

Implementation and evaluation recommendations for research translation programs***:*** In addition to the refined research translation theory, a Delphi Survey of experts will be undertaken to determine implementation and evaluation recommendations for research translation within the WA health system into the future. The survey will be conducted across several rounds via online questionnaire, which will be delivered until consensus is reached. The Delphi panel will consist of those involved in delivering the RTP program at the WA Department of Health, investigators involved in individual projects and other stakeholders[51,52]. These recommendations will include an evaluation framework specifically for the RTP program to be addressed by future funding recipients and selection panels.

Ethics and Dissemination: Curtin University Human Research Ethics Committee, Western Australia, approves this study (approval number: HRE2020-0464). Results will be published in scientific journals, and communicated to respondents and relevant partners.

For peer review only - http://bmjopen.bmj.com/site/about/guidelines.xhtml

REFERENCES

1. Elshaug AGP, Rosenthal MBP, Lavis JNP, et al. Levers for addressing medical underuse and overuse: achieving high-value health care. *Lancet* 2017;390(10090):191-202. doi: 10.1016/S0140-6736(16)32586-7
2. Braithwaite J, Zurynski Y, Ludlow K, et al. Towards sustainable healthcare system performance in the 21st century in high-income countries: a protocol for a systematic review of the grey literature. *BMJ Open* 2019;9(1) doi: 10.1136/bmjopen-2018-025892
3. Crisp N. What would a sustainable health and care system look like? *BMJ* 2017;358 doi: 10.1136/bmj.j3895
4. Sustainable Health Review. Sustainable Health Review: Final Report to the Western Australian Government. Western Australia.: Department of Health, 2019.
5. Braithwaite J, Testa L, Lamprell G, et al. Built to last? The sustainability of health system improvements, interventions and change strategies: a study protocol for a systematic review. *BMJ open* 2017;7(11):e018568-e68. doi: 10.1136/bmjopen-2017-018568
6. Tricco AC, Ashoor HM, Cardoso R, et al. Sustainability of knowledge translation interventions in healthcare decision-making: a scoping review. *Implement Sci* 2016;11(1):55-55. doi: 10.1186/s13012-016-0421-7
7. Grimshaw JM, Eccles MP, Lavis JN, et al. Knowledge translation of research findings. *Implement Sci* 2012;7(1):50-50. doi: 10.1186/1748-5908-7-50
8. Cruz Rivera S, Kyte DG, Aiyegbusi OL, et al. Assessing the impact of healthcare research: A systematic review of methodological frameworks. *PLOS Medicine* 2017;14(8):e1002370. doi: 10.1371/journal.pmed.1002370
9. Wensing M, Grol R. Knowledge translation in health: how implementation science could contribute more. *BMC medicine* 2019;17(1):88. doi: 10.1186/s12916-019-1322-9
10. Government of Western Australia; Department of Health. Research Translation Projects 2019 [Available from: https://ww2.health.wa.gov.au/Articles/N_R/Research-Translation-Projects.
11. Tricco AC, Ashoor HM, Cardoso R, et al. Sustainability of knowledge translation interventions in healthcare decision-making: a scoping review. *Implementation science : IS* 2016;11(55):55-55. doi: 10.1186/s13012-016-0421-7
12. Patsopoulos NA. A pragmatic view on pragmatic trials. *Dialogues Clin Neurosci* 2011;13(2):217-24.
13. Stirman S, Kimberly J, Cook N, et al. The sustainability of new programs and innovations: a review of the empirical literature and recommendations for future research. *Implement Sci* 2012;7(1) doi: 10.1186/1748-5908-7-17
14. Gruen RL, Elliott JH, Nolan ML, et al. Sustainability science: an integrated approach for health-programme planning. *The Lancet* 2008;372(9649):1579-89. doi: 10.1016/S0140-6736(08)61659-1
15. Scheirer MA. Is Sustainability Possible? A Review and Commentary on Empirical Studies of Program Sustainability. *The American journal of evaluation* 2005;26(3):320-47. doi: 10.1177/1098214005278752
16. Shelton RC, Cooper BR, Stirman SW. The Sustainability of Evidence-Based Interventions and Practices in Public Health and Health Care. *Annu Rev Public Health* 2018;39(1):55-76. doi: 10.1146/annurev-publhealth-040617-014731
17. Whelan J, Love P, Pettman T, et al. Cochrane Update: Predicting sustainability of intervention effects in public health evidence: identifying key elements to provide guidance. *Journal of Public Health* 2014;36(2):347-51. doi: 10.1093/pubmed/fdu027
18. Braithwaite J, Ludlow K, Testa L, et al. Built to last? The sustainability of healthcare system improvements, programmes and interventions: a systematic integrative review. *BMJ open* 2020;10(6):e036453. doi: 10.1136/bmjopen-2019-036453

For peer review only - http://bmjopen.bmj.com/site/about/guidelines.xhtml

19. Lennox L, Maher L, Reed J. Navigating the sustainability landscape: a systematic review of sustainability approaches in healthcare. *Implement Sci* 2018;13(1) doi: 10.1186/s13012-017-0707-4
20. Flynn R, Scott SD. Understanding Determinants of Sustainability Through a Realist Investigation of a Large-Scale Quality Improvement Initiative (Lean): A Refined Program Theory. *Journal of nursing scholarship : an official publication of Sigma Theta Tau International Honor Society of Nursing* 2019 doi: 10.1111/jnu.12527
21. Chambers DA, Glasgow RE, Stange KC. The dynamic sustainability framework: addressing the paradox of sustainment amid ongoing change. *Implementation science : IS* 2013;8(1):117-17. doi: 10.1186/1748-5908-8-117
22. Hawe P, Shiell A, Riley T. Theorising Interventions as Events in Systems. *American Journal of Community Psychology* 2009;43(3-4):267-76. doi: 10.1007/s10464-009-9229-9
23. Innovative Care for Chronic Conditions : Building Blocks for Action. Geneva: Geneva : World Health Organization 2002.
24. Hedelin B. Complexity is no excuse. *Sustainability Science* 2019;14(3):733-49. doi: 10.1007/s11625-018-0635-5
25. Greenhalgh T, Macfarlane F, Barton-Sweeney C, et al. "If We Build It, Will It Stay?" A Case Study of the Sustainability of Whole-System Change in London. *Milbank Quarterly* 2012;90(3):516-47. doi: 10.1111/j.1468-0009.2012.00673.x
26. Braithwaite J, Churruca K, Long JC, et al. When complexity science meets implementation science: a theoretical and empirical analysis of systems change. *BMC medicine* 2018;16(1):63. doi: 10.1186/s12916-018-1057-z
27. Wong G, Greenhalgh T, Westhorp G, et al. Realist methods in medical education research: what are they and what can they contribute? *Medical Education* 2012;46(1):89-96. doi: 10.1111/j.1365-2923.2011.04045.x
28. Pawson R. The science of evaluation : a realist manifesto / Ray Pawson. London: London : SAGE 2013.
29. Greenhalgh T, Wong G, Jagosh J, et al. Protocol—the RAMESES II study: developing guidance and reporting standards for realist evaluation. *BMJ Open* 2015;5(8) doi: 10.1136/bmjopen-2015-008567
30. Pawson R. Realistic evaluation / Ray Pawson and Nick Tilley. London: London : SAGE 1997.
31. Geoff W, Gill W, Joanne G, et al. Quality and reporting standards, resources, training materials and information for realist evaluation: the RAMESES II project. *Health Services and Delivery Research* 2017;5(28) doi: 10.3310/hsdr05280
32. Rycroft-Malone J, Burton C, Wilkinson J, et al. Collective action for knowledge mobilisation: a realist evaluation of the Collaborations for Leadership in Applied Health Research and Care. *Health Services and Delivery Research* 2015;3(44) doi: 10.3310/hsdr03440
33. Nurjono M, Shrestha P, Lee A, et al. Realist evaluation of a complex integrated care programme: protocol for a mixed methods study. *BMJ Open* 2018;8(3) doi: 10.1136/bmjopen-2017-017111
34. Shearn K, Allmark P, Piercy H, et al. Building Realist Program Theory for Large Complex and Messy Interventions. *International Journal of Qualitative Methods* 2017;16(1) doi: 10.1177/1609406917741796
35. Plsek PE, Greenhalgh T. The challenge of complexity in health care. *BMJ* 2001;323(7313):625. doi: 10.1136/bmj.323.7313.625
36. Moore GF, Audrey S, Barker M, et al. Process evaluation of complex interventions: Medical Research Council guidance. *BMJ : British Medical Journal* 2015;350(mar19 6) doi: 10.1136/bmj.h1258
37. Pawson R, Boaz A, Grayson L, et al. Types and quality of social care knowledge. Stage two: Towards the quality assessment of social care knowledge. *ESRC UK Center for Evidence Based Policy and Practice: Working Paper* 2003;18:1-31.

For peer review only - http://bmjopen.bmj.com/site/about/guidelines.xhtml

38. Flynn R, Rotter T, Hartfield D, et al. A realist evaluation to identify contexts and mechanisms that enabled and hindered implementation and had an effect on sustainability of a lean intervention in pediatric healthcare. *BMC Health Serv Res* 2019;19(1):912-12. doi: 10.1186/s12913-019-4744-3
39. Maxwell JA. A realist approach for qualitative research: Sage 2012.
40. Nevedal AL, Reardon CM, Jackson GL, et al. Implementation and sustainment of diverse practices in a large integrated health system: a mixed methods study. *Implementation Science Communications* 2020;1(1):61-61. doi: 10.1186/s43058-020-00053-1
41. Luck K. A proposed program theory for a smoke-free hospital property. *Soc Theory Health* 2017;15(3):323-45. doi: 10.1057/s41285-017-0031-8
42. Manzano A. The craft of interviewing in realist evaluation. *Evaluation* 2016;22(3):342-60. doi: 10.1177/1356389016638615
43. Greenhalgh T, Wong G, Westhorp G, et al. Protocol--realist and meta-narrative evidence synthesis: evolving standards (RAMESES). *BMC Med Res Methodol* 2011;11(1):115-15. doi: 10.1186/1471-2288-11-115
44. Pawson R, Greenhalgh T, Harvey G, et al. Realist review – a new method of systematic review designed for complex policy interventions. *J Health Serv Res Policy* 2005;10(1_suppl):21-34. doi: 10.1258/1355819054308530
45. Jagosh J. Realist Synthesis for Public Health: Building an Ontologically Deep Understanding of How Programs Work, For Whom, and In Which Contexts. *Annu Rev Public Health* 2019;40(1):361-72. doi: 10.1146/annurev-publhealth-031816-044451
46. Grol R, Wensing M, Eccles M, et al. Improving patient care: the implementation of change in health care2013.
47. Gilmore B, McAuliffe E, Larkan F, et al. How do community health committees contribute to capacity building for maternal and child health? A realist evaluation protocol. *BMJ Open* 2016;6(11):e011885-e85. doi: 10.1136/bmjopen-2016-011885
48. Byng R, Norman I, Redfern S. Using Realistic Evaluation to Evaluate a Practice-level Intervention to Improve Primary Healthcare for Patients with Long-term Mental Illness. *Evaluation (London, England 1995)* 2005;11(1):69-93. doi: 10.1177/1356389005053198
49. Marchal B, van Belle S, van Olmen J, et al. Is realist evaluation keeping its promise? A review of published empirical studies in the field of health systems research. *Evaluation (London, England 1995)* 2012;18(2):192-212. doi: 10.1177/1356389012442444
50. Salter KL, Kothari A. Using realist evaluation to open the black box of knowledge translation: a state-of-the-art review. *Implement Sci* 2014;9(1):115-15. doi: 10.1186/s13012-014-0115-y
51. Boulkedid R, Abdoul H, Loustau M, et al. Using and reporting the Delphi method for selecting healthcare quality indicators: a systematic review. *PLoS One* 2011;6(6):e20476-e76. doi: 10.1371/journal.pone.0020476
52. Hasson F, Keeney S, McKenna H. Research guidelines for the Delphi survey technique. *J Adv Nurs* 2000;32(4):1008-15. doi: 10.1046/j.1365-2648.2000.01567.x

Authors ‘contribution: AM, DH, EG, YZ, SR jointly conceived the study; AM and DH developed the study proposal; AM led the writing of this paper; DH, EG, YZ and SR read and reviewed the final version prior to submission.

For peer review only - http://bmjopen.bmj.com/site/about/guidelines.xhtml

Funding statement: This work is supported by the National Health and Medical Research Council Partnership Centre for Health System Sustainability (ID: 9100002). In addition the Western Australian Department of Health provides financial support for the Research Translation Projects program.

Competing interests: None declared.

Patient consent: Not required.

Provenance and peer review: Not commissioned; peer reviewed for ethical and funding approval prior to submission.

PPI statement:

Word count: 3,874 words.

For peer review only

For peer review only - http://bmjopen.bmj.com/site/about/guidelines.xhtml
